# Passive thigh heating improves peak force production in younger adults and early isokinetic force production in younger and older adults

**DOI:** 10.1113/EP092690

**Published:** 2025-11-10

**Authors:** Desmond Denny, Daniel C. Low, Oliver R. Gibson

**Affiliations:** ^1^ Department of Sport, Health and Exercise Sciences Brunel University of London Uxbridge UK; ^2^ Centre for Physical Activity in Health and Disease (CPAHD) Brunel University of London Uxbridge UK

**Keywords:** force development, hyperthermia, muscle strength, older adult, passive heating, thermal therapy, torque

## Abstract

Older adults often suffer from reduced physical capability relative to young adults, in part due to impaired muscle function. This study investigated the ergogenic effects of passive thigh heating on knee extensor torque production in healthy older versus younger adults. Twenty‐two younger (YOUNGER; 23 ± 3 years) and 16 older (OLDER; 68 ± 8 years) adults completed an experimental visit whereby one thigh was heated via a garment circulating 50°C water for 90 min (HEAT) with the contralateral limb unheated (CONT). Four maximal contractions were performed at three isokinetic speeds (slow, 60°/s; moderate, 180°/s; and fast, 300°/s) and an isotonic set (25% maximal voluntary isometric contraction force); contractions were performed on both limbs at baseline and every 30 min thereafter for 120 min, with the final time point used to quantify the retention/decay in response. Vastus lateralis temperature was measured every 30 min, and surface electromyography was implemented throughout. HEAT increased muscle temperature from baseline (31.7 ± 1.7°C) at 30 min (36.5 ± 1.5°C), peaking at 90 min (37.5 ± 0.7°C), all *P* < 0.05. HEAT increased peak torque during moderate (+11 ± 12 N m) and fast (+7 ± 11 N m) contractions in only YOUNGER participants relative to their control leg which remained unchanged (*P* < 0.05). After 30 min, rate of force development (RFD) in HEAT increased during slow contractions from baseline in both age groups (+229 ± 210 N m s^−1^, *P* < 0.05) and early force production (EFP) increased in both age groups during slow contractions from 60 min in HEAT (+15 ± 15 N m, *P* < 0.05). Peak EMG amplitude was unchanged throughout. Despite a similar increase in the RFD and EFP in both young and older adults, passive thigh heating improves peak knee extensor torque in moderate and fast isokinetic contractions in young adults only.

## Abstract (highlights)

- **What is the central question of this study?**
  Can passive thigh heating improve peak torque and early force production in younger and older adults?
- **What is the main finding and its importance?**
  Passive thigh heating increased skeletal muscle temperature and peak torque during moderate and fast isokinetic contractions in younger adults and improved early force production during slow contractions in both age groups. Improvements in peak torque decayed after 30 min post‐heating whilst improvements in early force production remained. These findings indicate that passive heating could enhance muscle function, especially in older adults where improved early force production may aid mobility and reduce fall risk.

## 1 INTRODUCTION

Muscle strength and power predicate effective human function and contribute to a higher quality of life. For older adults, muscular strength supports locomotion (Anderson et al., 2007) and facilitates postural control (Topp et al., 1997), and a higher level of muscular strength has been correlated to a reduced risk of falling (Pizzigalli et al., 2011). Further to this, maintaining muscular power can facilitate independent living, maintain a higher quality of life during ageing, and also reduce fall risk (Cruz‐Jentoft et al., 2010; Freitas et al., 2024; Gray & Paulson, 2014; Rice & Keogh, 2009). The decline of muscle function with increased age is often observed from 40 years onwards (McGregor et al., 2014) and is attributed to a loss of muscle mass, degradation of neural pathways, decreases in mitochondrial function and chemical handling, and reductions in hormone levels (Cruz‐Jentoft & Sayer, 2019; Cruz‐Jentoft et al., 2010; Morley et al., 2001). These changes in muscle physiology are commonly quantified via observed reductions in maximal force production (greatest amount of force a muscle or group of muscles can generate in a single voluntary contraction, a proxy for maximal strength; Tøien et al., 2025) and slower rates of force production (a proxy for muscle power; Barry et al., 2005; Thompson et al., 2013). In conjunction with general age‐related declines in muscular strength and power production, older adults are often exposed to prolonged periods of inactivity, which can contribute to a reduced muscle temperature (*T*_mu_) (Gibson et al., 2023). Skeletal muscle has been reported to perform optimally during physical activity at temperatures above the resting physiological range of 32–35°C (Bishop, 2003), with hyperthermic skeletal muscle displaying increases in force production relative to normothermic tissue (Ranatunga, 1984; Sargeant, 1987). Whilst an increase in *T*_mu_ is normally achieved via physical activity, for example, through a traditional active warm‐up (Racinais et al., 2017), for some populations, for example, those who have developed frailty and immobility, this is impractical (Strandberg et al., 2011).

Acute increases in *T*_mu_ are associated with a variety of physiological changes, including, but not limited to, an increase in localised blood flow into and out of the heated muscle (Chiesa et al., 2015; Watanabe et al., 2024), improved myofibrillar calcium handling (Kobayashi et al., 2005), altered muscle–tendon stiffness (Rodrigues et al., 2022), optimised penetration angle (Eng et al., 2018), and increases in ATP turnover and muscle fibre conduction velocity (Gray et al., 2006). Experimental literature investigating functional outcomes associated with non‐physical activity‐related increases in skeletal *T*_mu_ in young adults has observed increases in early force production (EFP; i.e., absolute force produced at 0.18 s) and rate of force development at 50 ms (RFD_50_; i.e., the rate at which a muscle develops force during the first 50 ms of contraction) during isometric and slow (60°/s) isokinetic contractions (Denny et al., 2025; Rodrigues et al., 2021), increases in peak torque during moderate and fast isokinetic contractions (Denny et al., 2025), and improvements in force production and/or performance during functional movements such as jumping (Skurvydas et al., 2008) and cycle sprinting (Sargeant, 1987). RFD_50_ has been identified as a factor in postural control and balance in elderly men and women (Ema et al., 2016), as well as a predictor of faster timed up and go test scores, which in turn indicates higher levels of functional mobility (Hester et al., 2021). Research indicates that EFP (e.g., force produced within 50–200 ms) is crucial for real‐world tasks like recovering from a stumble or initiating movement quickly (Bellumori et al., 2013; Maffiuletti et al., 2016; Orssatto et al., 2020), and is therefore both a measure of absolute force production at 0.18 s, and when combined with RFD_50_ captures complementary but distinct aspects of neuromuscular performance during initial contractile phases. Taken together, it has been proposed that an acute increase in muscle function across the aforementioned contraction types and facilitated by passively increased *T*_mu_ may aid physical activity and the performance of everyday tasks in cohorts with sub‐optimal muscle function, for example, older adults. To date, however, investigation into the efficacy of passive heating of skeletal muscle as a means to improve contractile function across multiple contraction types and velocities in older adults is limited. Critically, whether the intervention has efficacy beyond cohorts of young adults also remains unknown. Should observations regarding the ergogenic benefit of passive heating in younger adults also be observed in older cohorts, passive heating may be considered a viable intervention to enhance the ability of older adults to independently complete daily living tasks (Hortobágyi et al., 2003; Katula et al., 2008; Khodadad Kashi et al., 2023).

Whilst prior work in young adults has identified passive heating as a potential ergogenic aid to improve muscle function, the dose–response relationship is also yet to be effectively characterised. Additionally, whilst it is understood that maintaining increased *T*_mu_ is important for maintaining peak force production (Faulkner et al., 2013), the retention and/or decay following passive heating cessation have yet to be fully quantified. Beyond the physiological and functional benefits, the study of perceptual effects following passive heating has been restricted to pain management (Chabal et al., 2020). The discomfort associated with physical activity is inversely associated with enjoyment or pleasure when partaking in physical activity or exercise (Ekkekakis & Petruzzello, 2012; Ekkekakis et al., 2005). Therefore, understanding whether heating improves these sensations, that is, perceived effort and feelings of readiness to participate in physical activity, is noteworthy in the context of exercise initiation and adherence. Perceptions of self‐ability have been identified as barriers to physical activity for older adults (Rúa‐Alonso et al., 2023). A simple heating intervention may help increase confidence and improve perceptions of readiness for exercise. It is also important that participants believe that the intervention is beneficial for adherence or usage of the tool if it is to be implemented.

The primary aim of this study was to quantify the effect of 90 min of passive thigh heating on peak torque and EFP and on RFD_50_ during isokinetic contractions in younger versus older adults. The secondary aim of the study was to measure the retention or decay of the anticipated improvements in isokinetic peak torque and EFP and RFD_50_ in younger versus older adults 30 min post‐cessation of heating. The final aim was to examine the perceptual responses to the heating protocol and quantify how both younger and older adults perceived passive heating to influence muscle function. It was hypothesised that passive heating would enhance peak isokinetic torque at moderate and fast contractile speeds, whilst also improving RFD and early force production at the slowest contractile speed in both younger and older adults. These enhancements were expected to persist for up to 30 min following the heating intervention. Additionally, both age groups were anticipated to perceive passive heating as beneficial to muscle function.

## 2 METHODS

### 2.1 Ethical approval

The study was approved by the Brunel University of London Research Ethics Committee (44577‐MHR‐Oct/2023‐47671‐3) and was carried out in accordance with the *Declaration of Helsinki*. Written informed consent was obtained from all participants prior to commencement of the study.

### 2.2 Participants

Twenty‐two active (>60 min of self‐reported physical activity per week) younger adults (YOUNGER) and 16 older adults (OLDER), who were at least recreationally active and capable of self‐locomotion, completed the study. All participants were non‐smokers, with no history of heat intolerance or neuromuscular disorders. OLDER completed the WHOQOL‐BREF questionnaire (von Steinbüchel et al., 2006) with all participants indicating a ‘high quality of life’ relative to the international validation study of the WHOQOL‐BREF (Skevington et al., 2004). Older adults were excluded from participation if they were taking medication that thinned the blood or was an anti‐coagulant. The target sample size was estimated from a prior experimental study involving young adults only (Denny et al., 2025). An effect size of 0.6 was calculated for the difference in torque produced between the heated condition and control condition within the analysis. Therefore, in conjunction with an α set to 0.05 and β at 0.8, a sample minimum size of at least 16 participants per group was required. A subset of the YOUNGER participants had previously undertaken an associated reliability experiment (Denny et al., 2025). Due to the single‐visit design, the menstrual phase was not controlled for in the YOUNGER female cohort, and OLDER females were all post‐menopausal. Participant descriptive characteristics are presented in Table 1.

**TABLE 1:** Participant characteristics for younger (YOUNGER) and older (OLDER) adults.

	YOUNGER	OLDER
Sex (male/female)	12/10	8/8
Age (years)	23 ± 3	68 ± 8^a^
Height (m)	1.72 ± 0.80	1.65 ± 0.10
Mass (kg)	68.5 ± 12.5	67.7 ± 13.1
BMI (kg/m^2^)	23.1 ± 3.3	24.8 ± 4.4
Body fat (%)	17 ± 4	27 ± 4^a^
Peak isometric force HEAT (N m)	238 ± 70 ^a^, ^b^	153 ± 82^a^, ^b^
Peak isometric force CONT (N m)	201 ± 65	124 ± 78

Values are means ± SD.

^a^ Significant difference in mean between YOUNGER (*n* = 22) and OLDER (*n* = 16).

^b^ Significant difference between heated (HEAT) and control (CONT) limb. BMI, body mass index.

### 2.3 Experimental design

**FIGURE 1:**
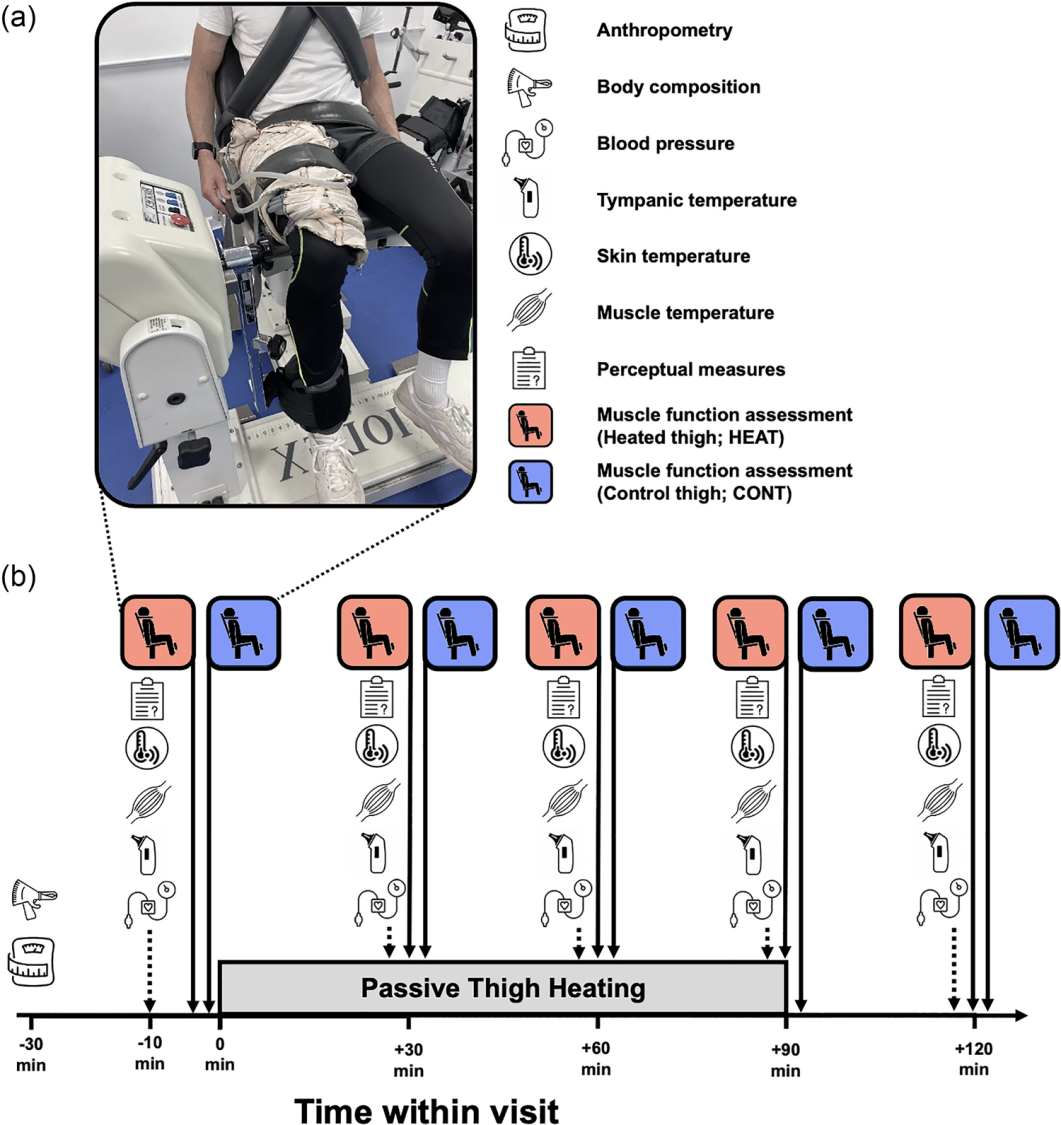
(a) Image of the custom‐made water‐perfused heated garment as affixed on the thigh without the covering of the survival blanket. (b) Sequence of the experimental protocols.

Participants visited the laboratory (ambient room temperature YOUNGER = 19 ± 2°C, OLDER = 19 ± 2°C) for a single visit either at 09.00 or 13.00 h having abstained from heavy exercise (e.g. resistance training, prolonged endurance activity or competitive sport), caffeine and alcohol for 24 h prior to the experimental visits, with adherence verbally confirmed by the participants upon arrival. The visit began with an assessment of anthropometric characteristics. Body fat percentage was determined in accordance with the Durnin and Womersley (1977) four‐site skinfold method (YOUNGER) or following 15 min of rest via bioelectrical impedance (OLD; Bodystat 1500, Bodystat Ltd, Douglas, Isle of Man, UK). These two measures have been demonstrated as comparable, with error trending in the direction of bioelectrical impedance underestimating skinfold values (Silveira et al., 2020). Unshod standing stature was recorded using a stadiometer (SECA model 213, Hamburg, Germany), with mass was assessed using electronic scales (SECA model 875, Hamburg, Germany). Physiological and perceptual measures were assessed first, followed by muscle function outcomes. These measures were taken at baseline (0 min), then +30, +60, +90 and +120 min thereafter on both limbs. Following instrumentation, the right leg (60% of participants’ dominant limb) was prepared to have the upper thigh heated for 90 min (Heat) whilst the contralateral limb served as a control (Cont). Contralateral limb testing has been identified as an effective, robust and efficient method of observing muscle responses to a range of interventions (Macinnis et al., 2017). The participants wore leggings with the experimental thigh (Heat) wrapped in a custom garment that circulated water at an outlet temperature of 50°C and a survival blanket for a period of 90 min. The garment and survival blanket remained on the limb for the entire testing protocol, including during muscle function assessment. The contralateral control thigh (Cont) was left uncovered. The implemented water temperature was utilised based upon pilot and experimental work that had examined safety, efficacy and tolerability in groups of younger and older adults (Gibson et al., 2023; Koch Esteves et al., 2021, 2023; Watanabe et al., 2024). The participants remained seated on the dynamometer throughout the testing protocol. Whilst not engaged in knee extensor exercise, the participants had their feet resting on a chair with their knees bent at a ∼90° angle. All contractions were conducted through a 75° to 175° range of motion. Figure 1 provides a schematic overview of the water‐perfused garment (a) and experimental trial timeline (b).

### 2.4 Physiological and perceptual measures

A tympanic membrane temperature device (Brawn Thermoscan 7, Bussigny, Switzerland) was set to the appropriate age and then fully inserted into the right ear canal, whereby tympanic temperature (*T*_tymp_) was recorded. *T*_tymp_ is considered a valid surrogate for a direct measure of deep body (core) temperature in the rested state across a range of indoor environmental conditions, including the room temperature associated with this experiment (Sato et al., 1996). Heart rate (HR), systolic (SBP) and diastolic (DBP) blood pressure, and mean arterial pressure (MAP) were measured via an automated sphygmomanometer placed over the left brachial artery (Carescape V100 VitalSigns Monitor, Bolton, UK). To measure *T*_mu_, a muscle temperature probe (T‐204f, PhysiTemp, NJ, USA) was connected to a portable digital thermometer (RS 103‐433 K‐type thermocouple unit, Corby, UK) and inserted, without local anaesthesia, ∼30 mm below the skin surface at a 45° angle to the horizontal into the vastus lateralis via an 18‐gauge hypodermic needle (Microlance 3, Fraga, Spain). Participant's *T*_mu_ was manually recorded following temperature stabilisation (typically ∼5 s) with the probe and guide needle removed thereafter. The insertion depth (∼30 mm) was chosen to minimize the influence of subcutaneous fat thickness and skin surface cooling, which can lead to underestimation of intramuscular temperature if the probe is positioned too superficially. This methodological concern regarding measurement depth has been highlighted in recent work examining the accuracy of thermocouple placement within muscle tissue (Rodrigues et al., 2024). Wireless iButton (DS1922L Thermochron Data Logger, Maxim/Dallas Semiconductor Corp, CA, USA) sensors were placed on the muscle belly of the vastus lateralis and used to measure thigh skin temperature (*T*_skin_) at 1‐min intervals. Participants responded to a global rate of change scale (+7 a very great deal better, +4 moderately better, 0 about the same, −4 moderately worse, −7 a very great deal worse; Bobos et al., 2020) and thermal sensation scale (0.0 unbearable cold, 2.0 cold, 4.0 neutral (comfortable), 6.0 hot, 8.0 unbearably hot; Young et al., 1987) prior to assessment of muscle function. A rate of perceived exertion scale (RPE; 6 no exertion; 7 very, very light; 13 somewhat hard; 19 very, very hard; 20 maximal exertion; Borg, 1990) was shown and answered by participants after every set of knee extension at 60°/s.

### 2.5 Muscle function and surface electromyography

Following the assessment of physiological measures, knee extensor function was assessed using a dynamometer (Biodex Medical Systems, Shirley, NY, USA). A warm‐up of 10 submaximal knee extensions (five at 50% maximum effort, three at 75% maximum effort, two at 90% of maximum effort, conducted on each leg at a self‐selected intensity) was followed by an assessment of maximal voluntary isometric contraction force (MVIC) on both limbs. At baseline (0 min), and +30, +60, +90 and +120 min thereafter, participants performed four repetitions of maximal isokinetic knee extension at 60°/s, 180°/s and 300°/s, separated by 60 s of passive rest, then performed four isotonic contractions against 25% of their MVIC. Muscle function testing was conducted in accordance with previous work investigating isokinetic (Blazquez et al., 2013) and isotonic muscle (Cheng & Rice, 2005). At all time points, the HEAT limb was assessed in full first, followed by Cont. The single highest, that is, the peak recorded torque value of each set, was used independently for torque analysis, unless stated, across all contraction types at every time point. In addition to peak torque, measures of early muscular force and power production were recorded, as prior research indicates that rapid force and power development are severely diminished with ageing (Bellumori et al., 2013; Crozara et al., 2013). Prior research suggests that localised passive heating can increase RFD_50_ in younger adults (Mornas et al., 2022; Rodrigues et al., 2021). The RFD_50_ was calculated as the first positive torque data point subtracted from the torque value at 50 ms after the first recorded value, which was then divided by the time elapsed in seconds (Maffiuletti et al., 2016). The RFD_50_ was calculated for repetitions at 60°/s. Peak force produced at 0.18 s was recorded as a measure of EFP, with EFP recorded during the four repetitions at each velocity (i.e., at 60°/s (EFP_60_) and 180°/s (EFP_180_)). Contractions at 300°/s and the isotonic contractions at 25% MVIC were not reported for EFP as the OLDER group were not all able to produce measurable force at 0.18 s. The peak torque value of each set was analysed across all contraction types at every time point. The EFP was calculated as peak force produced at 0.18 s during the four repetitions produced during contractions at 60°/s (EFP_60_) and at 180°/s (EFP_180_). The Biodex System 4 software was used to collect data at 100 Hz. Torque, position and velocity data were therefore collected within the software every 10 ms, then exported without filtering and imported to Microsoft Excel for analysis. EMG acquisition software (Delsys Discover, Boston, MA, USA) was used to collect the raw EMG data from the EMG sensors (Delsys Trigno). The data were plotted within the acquisition software before being exported. Once exported, the data were processed with custom MATLAB (MathWorks, Natick, MA, USA) code. Raw EMG signals were bandpass filtered with a 6–450 Hz cut‐off frequency in accordance with similar studies (Gordon et al., 2023) before subtracting the mean of the signal to correct baseline offsets. The filtered signal then underwent full‐wave rectification and low‐pass filtering to produce a linear envelope using a dual‐pass second‐order Butterworth filter, after which peak amplitude within each set was reported.

### 2.6 Statistical analysis

All data were analysed using SPSS Statistics software (Version 25; IBM Corp., Armonk, NY, USA). Normality was assessed using the Shapiro–Wilk test, which showed that some variables were normally distributed whilst others were not. Despite this, ANOVA was used due to its robustness to moderate violations of normality. Sphericity was evaluated using Mauchly's test, and when violated, the Greenhouse–Geisser correction was applied. A three‐way repeated‐measures ANOVA was used to determine main effect differences across time points (0, +30, +60, +90 min), between conditions (HEAT and CONT) and between groups (YOUNGER, OLDER) for muscle function measures, *T*_mu_, *T*_skin_ and RPE. For all other experimental variables, a two‐way ANOVA mixed model was used to determine main effect differences across time points (0, +30, +60, +90 min), and between groups (YOUNGER, OLDER). A two‐way ANOVA mixed model was also used to determine main effect differences in peak isometric force between conditions (HEAT, CONT) and groups (YOUNGER, OLDER). An independent sample Student's *t*‐test was used to determine differences in participant characteristics between groups. Bonferroni *post hoc* adjusted pairwise comparisons were used where significant main effects occurred to identify interaction effects between individual time points between conditions and visits. Statistical significance was set at *P* < 0.05, data are reported Mean ± SD. Global rate of change scales had results manually counted and reported as the frequency of responses. Effect size was calculated using partial eta squared (η_p_^2^) and was graded to be a small effect size if η_p_^2^ > 0.01, a medium effect size if η_p_^2^ > 0.06 and a large effect size if η_p_^2^ > 0.14 (Fritz et al., 2012).

## 3 RESULTS

### 3.1 Participant characteristics

By design, between‐group differences were observed for age, with greater body fat also observed in the OLDER group. Participants were successfully matched for all other anthropometric characteristics. Peak isometric force analysis revealed an age × condition interaction (*F*_(1,35)_ = 9.3, *P* = 0.004, η_p_^2^ = 0.21) whereby significantly greater force was observed in YOUNGER versus OLDER in both HEAT (+86 ± 144 N m, +43 ± 58%) and CONT (+75 ± 134 N m, +47 ± 60%). Further to this, differences between HEAT and CONT were observed for YOUNGER (+30 ± 13 N m, +17 ± 7%) and OLDER (+19 ± 16 N m, +21 ± 18%). See Table 1 for all participant characteristic data.

### 3.2 Systemic physiological and local temperature responses to passive thigh heating

**FIGURE 2:**
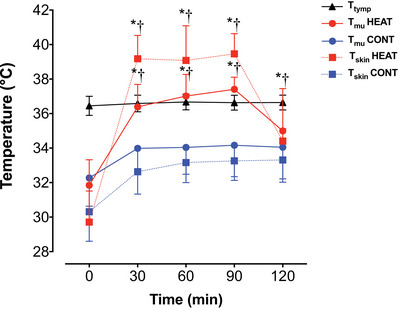
Mean ± SD change in muscle temperature (*T*_mu_, circles), thigh skin temperature (*T*_skin_, squares) and tympanic temperature (*T*_tymp_, triangles) during the passive thigh heating protocol (*T*_tymp_ and *T*_skin_ *n* = 29, *T*_mu_ *n* = 13).*Significant difference between HEAT (red) and CONT (blue) at corresponding time point (*P* < 0.05). †Significant difference from 0 min within condition.

The *T*_mu_ analysis revealed an effect of condition (*F*(_1,11)_ = 23.5, *P* < 0.001, η_p_^2^ = 0.27), with the effect of time also significant (*F*_(1,44)_ = 36.7, *P* < 0.001, η_p_^2^ = 0.77). Most noteworthy is the significant interaction effects of condition and time (*F*_(4,44)_ = 18.4, *P* < 0.001, η_p_^2^ = 0.63) whereby significant increases in *T*_mu_ from baseline were detected in HEAT at 30 min (+4.3 ± 1.9°C), 60 min (+4.9 ± 1.7°C), 90 min (+5.3 ± 1.6°C) and 120 min (+2.4 ± 2.3°C). A significant increase in *T*_mu_ was observed in CONT from baseline, at 30 min (+1.6 ± 1.1°C), 60 min (+1.8 ± 1.6°C) and 90 min (+1.8 ± 1.8°C); however, the change was no longer significant at 120 min (+1.8 ± 1.9°C). Muscle temperature in HEAT was not different at baseline, but significantly higher than CONT at 30 min (+2.4 ± 1.9°C), 60 min (+2.9 ± 1.6°C) and 90 min (+3.3 ± 2.0°C); no significant difference was seen at 120 min (+0.4 ± 1.9°C). Skin temperature showed significant differences when considering the main effects of condition (*F*_(1,24)_ = 80.0, *P* < 0.001, η_p_^2^ = 0.77); the effect of time was also significant (*F*_(4,96)_ = 30.8, *P* < 0.001, η_p_^2^ = 0.56). The interaction effect between condition and time is most relevant (*F*_(4,96)_ = 24.2, *P* < 0.001, η_p_^2^ = 0.50) whereby no significant differences were observed at baseline between HEAT and CONT, but significant increases in HEAT from baseline were observed at 30 min (+10.1 ± 3.0°C), 60 min (+10.4 ± 4.0°C), 90 min (+9.8 ± 2.3°C) and 120 min (+5.5 ± 4.8°C). CONT saw no significant changes from baseline. There was no difference between YOUNGER and OLDER in any temperature response, with combined temperature data presented in Figure 2. Mean arterial pressure differed when the main effects of time was observed (*F*_(4,140)_ = 6.1, *P* < 0.001, η_p_^2^ = 0.15); a significant change of −5 mmHg was observed at 60 min and of −8 mmHg at 90 min. No main or interaction effects were found when observing *T*_tymp_ (grand mean 36.3 ± 1.1°C) or heart rate (grand mean 75 ± 10 beats min^−1^). Results were not different between conditions at any time point and did not differ between YOUNGER and OLDER. Full *post hoc* comparisons can be found in Table 2 with all statistical outcomes related to these variables presented in Table A1.

**TABLE 2:** Physiological and perceptual responses to 90 min of passive thigh heating and 30 min following heating withdrawal in YOUNGER (*n* = 22) and OLDER (*n* = 16), muscle temperature in YOUNGER (*n* = 10) and OLDER (*n* = 3) and skin temperature in YOUNGER (*n* = 15) and OLDER (*n* = 14).

	0 min	30 min	60 min	90 min	120 min
**YOUNGER**					
Heart rate (beats min^−1^)	75 ± 14	81 ± 13	81 ± 11	81 ± 13	82 ± 13
*T*_mu_ (HEAT) (°C)^c^	31.7 ± 1.7	36.5 ± 1.5	37.1 ± 1.4	37.5 ± 0.7	35.5 ± 2.3
*T*_mu_ (CONT) (°C)	32.3 ± 1.8	34.1 ± 1.5	34.1 ± 1.8	34.3 ± 2.1	34.0 ± 2.0
*T*_skin_ (HEAT) (°C)^c^	28.9 ± 1.5	39.4 ± 0.9	39.0 ± 2.6	39.6 ± 1.2	34.3 ± 0.8
*T*_skin_ (CONT) (°C)	30.1 ± 1.9	32.7 ± 1.7	33.3 ± 1.4	33.3 ± 1.1	33.3 ± 1.
*T*_tymp_ (°C)	36.7 ± 0.6	36.8 ± 0.4	36.9 ± 0.4	36.8 ± 0.4	36.7 ± 0.5
MAP (mmHg)	91 ± 12	86 ± 8	86 ± 8	85 ± 8	88 ± 7
RPE (HEAT)^c^	13 ± 2^a^	15 ± 1^a^, ^b^	15 ± 2^a^, ^b^	16 ± 2^a^, ^b^	16 ± 2^a^, ^b^
RPE (CONT)	15 ± 1	16 ± 1	16 ± 2	17 ± 2	17 ± 2
Thermal sensation	3.9 ± 0.9	5.0 ± 0.6^b^	5.2 ± 0.7^b^	5.2 ± 0.9^b^	4.0 ± 0.8^b^
**OLDER**					
Heart rate (b.min^−1^)	69 ± 13	71 ± 13	71 ± 11	69 ± 10	70 ± 11
*T*_mu_ (HEAT) (°C)^c^	32.2 ± 0.8	36.1 ± 0.4	36.7 ± 0.5	37.2 ± 0.7	33.3 ± 2.6
*T*_mu_ (CONT) (°C)	32.2 ± 1.0	33.6 ± 1.1	33.9 ± 0.8	33.8 ± 0.6	34.1 ± 1.4
*T*_skin_ (HEAT) (°C)^c^	30.5 ± 1.7	38.9 ± 1.7	39.2 ± 1.1	39.3 ± 1.2	34.5 ± 0.7
*T*_skin_ (CONT) (°C)	30.6 ± 1.5	32.6 ± 1.1	33.1 ± 1.0	33.2 ± 1.2	33.3 ± 1.5
*T*_tymp_ (°C)	36.2 ± 0.4	36.3 ± 0.4	36.4 ± 0.4	36.4 ± 0.3	36.5 ± 0.3
MAP (mmHg)	102 ± 11	98 ± 9	97 ± 13	93 ± 12	99 ± 21
RPE (HEAT)^c^	15 ± 1	14 ± 2	15 ± 2	15 ± 2	15 ± 2
RPE (CONT)	14 ± 1	15 ± 2	16 ± 2	15 ± 2	15 ± 2
Thermal sensation	3.7 ± 1.0	4.7 ± 0.7^b^	4.7 ± 0.9^b^	5.0 ± 1.1^b^	4.5 ± 1^b^
**All participants**					
Heart rate (b.min^−1^)	74 ± 11	81 ± 9	81 ± 9	80 ± 10	76 ± 9
*T*_mu_ (HEAT) (°C)^c^	32.0 ± 1.2	36.6 ± 0.9^a^, ^b^	36.9 ± 1.0^a^, ^b^	37.3 ± 0.7^a^, ^b^	34.4 ± 2.4^b^
*T*_mu_ (CONT) (°C)	32.2 ± 1.4	33.8 ± 1.3^b^	34.0 ± 1.3^b^	34.0 ± 1.3^b^	34.1 ± 1.7
*T*_skin_ (HEAT) (°C)^c^	29.7 ± 1.6	39.2 ± 1.3^a^, ^b^	39.1 ± 1.9^a^, ^b^	39.5 ± 1.2^a^, ^b^	34.4 ± 0.7^a^, ^b^
*T*_skin_ (CONT) (°C)	30.3 ± 1.7	32.6 ± 1.4	33.2 ± 1.2	33.3 ± 1.2	33.3 ± 1.3
*T*_tymp_ (°C)	36.4 ± 0.5	36.6 ± 0.4	36.6 ± 0.4	36.6 ± 0.4	36.6 ± 0.4
MAP (mmHg)	97 ± 12	92 ± 9	92 ± 11	89 ± 10	94 ± 16
RPE (HEAT)^c^	14 ± 1^a^	15 ± 1^a^	15 ± 1^a^, ^b^	16 ± 1^a^, ^b^	15 ± 2^a^, ^b^
RPE (CONT)	14 ± 1	15 ± 2^b^	16 ± 2^b^	16 ± 2^b^	16 ± 2^b^
Thermal sensation	3.8 ± 0.7	4.9 ± 0.5^b^	5.0 ± 0.6^b^	5.1 ± 0.7^b^	4.3 ± 0.7

Data are means ± SD.

^a^ *P *< 0.05 versus control at corresponding time point.

^b^ *P *< 0.05 versus baseline (0 min).

^c^ Main effect for difference between conditions overall, *P *< 0.05.

### 3.3 Torque production following upper thigh muscle hyperthermia

**FIGURE 3:**
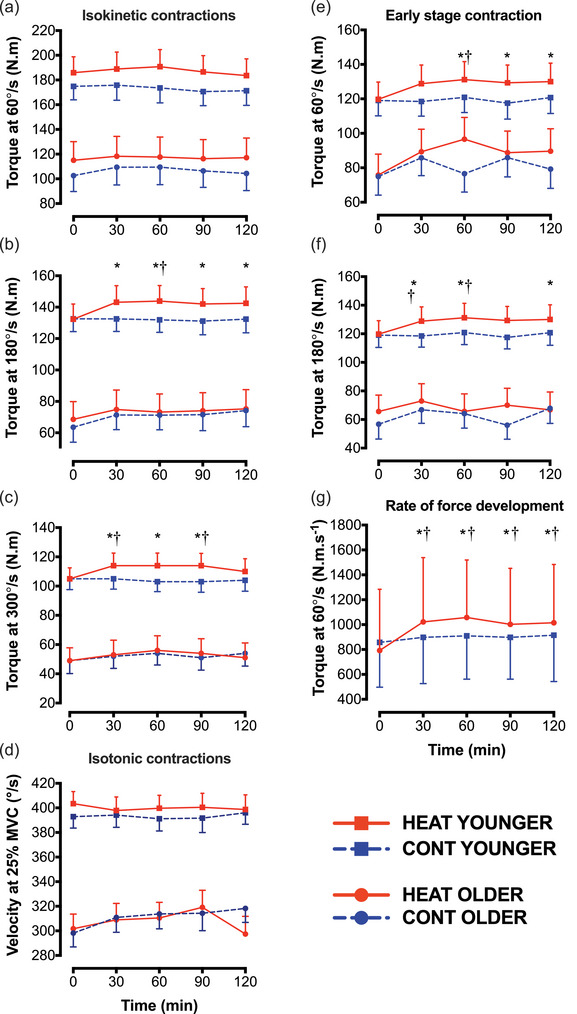
Mean ± SE peak torque production across 60°/s (a), 180°/s (b), 300°/s (c) and maximum velocity (d), early force production across 60°/s (e), 180°/s (f), and rate of force development in 60°/s (g) over 90 min of passive thigh heating and 30 min following heating withdrawal. Squares represent YOUNGER group and circles represent OLDER group, Red symbols represent HEAT, and blue symbols represent CONT. *Significant difference between HEAT and CONT at corresponding time point (*P* < 0.05). †Significant difference from baseline (*P* < 0.05).

YOUNGER produced significantly more force than OLDER at every isokinetic contractile speed (*P* < 0.001). When comparing the effect of heating on peak torque production, force production at 60°/s differed only between conditions (*F*_(1,36)_ = 13.3, *P* ≤ 0.001, η_p_^2^ = 0.27) with the HEAT leg producing +12 ± 21 N m (+9 ± 15%) more force. Differences were seen at 180°/s when observing the main effect of time (*F*_(1,144)_ = 5.7, *P* = 0.004, η_p_^2^ = 0.138) with an increase of +6 ± 11 N m seen at 30 and 60 min. When interpreting the time × condition × group interaction (*F*_(4,144)_ = 3.1, *P* = 0.018, η_p_^2^ = 0.079), YOUNGER produced a significant +11 ± 17 N m (+8 ± 12%) increase in peak torque from baseline in the HEAT versus CONT in all time points past baseline whilst the OLDER saw a non‐significant +5 ± 16 N m (+7 ± 20%) change from baseline after 30 min, +3 ± 17 N m (+5 ± 28%) at 60 min, +2 ± 15 N m (+3 ± 8%) at 90 min and +1 ± 19 N m (+2 ± 7%) at 120 min. Differences were seen at 300°/s when observing the main effect of condition (*F*_(1,36)_ = 5.9, *P* = 0.021, η_p_^2^ = 0.14); a significant increase in HEAT (+4 ± 9 N m) was seen. An interaction effect of condition × time (*F*_(4,144)_ = 2.8, *P* = 0.015, η_p_^2^ = 0.09) was observed, and whilst no difference was observed between conditions at baseline, significant differences were seen in HEAT at 30 min (+7 ± 2 N m; +8 ± 2%), at 60 min (+7 ± 2 N m; +8 ± 2%) and at 90 min (+6 ± 2 N m; +7 ± 2%) when compared to CONT. There was no significant difference between conditions in the decay measure at 120 min. HEAT significantly increased from baseline in YOUNGER at 30, 60 and 90 min by 8 ± 14 N m (+10 ± 11%); no significant changes occurred in CONT from baseline whilst the OLDER saw a non‐significant + 5 ± 12 N m (+10 ± 24%) change from baseline after 30 min, +7 ± 14 N m (+13 ± 16%) at 60 min, +6 ± 13 N m (+12 ± 26%) at 90 min and +2 ± 16 N m (+4 ± 8%) at 120 min. Peak velocity during the isotonic contractions at 25% MVIC had no main or interaction effects; results were not different between conditions at any time point, and did not differ between YOUNGER and OLDER (*P *> 0.05). Full *post hoc* comparisons can be found in Table 3 and Figure 3, 4 with all statistical outcomes related to peak torque presented in Table A2.

**TABLE 3:** Mean ± SD (lower, upper 95% confidence interval) peak torque and peak velocity (left) and early force production (torque at 0.18 s) and early velocity production (right) generated at 60°/s, 180°/s and 300°/s and versus 25% MVIC in heated (HEAT) and control (CONT) legs across 90 min of passive thigh heating and 30 min following heating withdrawal, YOUNGER (*n* = 22), OLDER (*n* = 16).

	0 min	30 min	60 min	90 min	120 min		0 min	30 min	60 min	90 min	120 min
YOUNGER											
Isokinetic contractions – peak torque (N m)						Isokinetic contractions – early force production (N m)					
60°/s (HEAT)	186 ± 50 (160, 212)	189 ± 54 (161, 217)	191 ± 54 (163, 219)	187 ± 52 (160, 213)	184 ± 53 (156, 211)	60°/s (HEAT)	120 ± 50 (99, 140)	129 ± 54^a^ (107, 151)	131 ± 52^a^, ^b^ (110, 152)	129 ± 51^a^ (108, 150)	130 ± 54^a^ (108, 152)
60°/s (CONT)	175 ± 43 (153, 197)	176 ± 48 (151, 201)	174 ± 48 (149, 198)	171 ± 45 (148, 194)	171 ± 47 (147, 195)	60°/s (CONT)	119 ± 44 (101, 137)	118 ± 42 (101, 136)	121 ± 44 (103, 139)	118 ± 46 (99, 136)	121 ± 46 (102, 140)
180°/s (HEAT)	132 ± 38 (113, 152)	143 ± 42 ^a^, ^b^ (122, 164)	144 ± 39^a^, ^b^ (124, 164)	142 ± 39^a^ (122, 162)	142 ± 41^a^ (121, 164)	180°/s (HEAT)	120 ± 46 (101, 139)	129 ± 49 (109, 149)	131 ± 50 (111, 152)	129 ± 48 (109, 149)	130 ± 51 (109, 151)
180°/s (CONT)	133 ± 32 (116, 149)	133 ± 32 (116, 149)	132 ± 32 (116, 148)	131 ± 35 (113, 149)	132 ± 35 (115, 150)	180°/s (CONT)	119 ± 42 (102, 136)	118 ± 38 (103, 136)	121 ± 41 (104, 134)	118 ± 40 (101, 134)	121 ± 43 (103, 139)
300°/s (HEAT)	105 ± 30 (90, 120)	114 ± 34 (96, 131)	114 ± 34 (96, 131)	114 ± 33 (97, 131)	111 ± 34 (93, 128)						
300°/s (CONT)	105 ± 30 (90, 120)	105 ± 28 (91, 120)	103 ± 27 (89, 117)	103 ± 29 (88, 118)	104 ± 30 (89, 119)						

Isotonic contractions – peak velocity (°/s)						Isokinetic contractions – RFD_50_					
25% MVIC (HEAT)^d^	403 ± 52 (383, 423)	398 ± 56 (376, 420)	400 ± 54 (378, 421)	400 ± 56 (377, 424)	399 ± 54 (375, 423)	60°/s (HEAT)	988 ± 660 (764, 1211)	1229 ± 684 (996, 1461)	1297 ± 612 (1088, 1503)	1255 ± 600 (1051, 1459)	1229 ± 630 (1017, 1442)
25% MVIC (CONT)^d^	393 ± 49 (374, 412)	394 ± 48 (374, 414)	391 ± 49 (371, 411)	392 ± 55 (378, 415)	396 ± 48 (377, 415)	60°/s (CONT)	1105 ± 486 (941, 1268)	1153 ± 498 (984, 1321)	1158 ± 462 (1001, 1315)	1128 ± 444 (977, 1279)	1145 ± 492 (977, 1312)

OLDER											
Isokinetic contractions – peak torque (N m)						Isokinetic contractions – early force production (N m)					
60°/s (HEAT)	115 ± 59 (88, 151)	118 ± 63 (89, 156)	118 ± 64 (88, 156)	116 ± 61 (88, 153)	117 ± 63 (88, 154)	60°/s (HEAT)	76 ± 57 (51, 100)	89 ± 62 (63, 116)	97 ± 60^a^, ^b^ (71, 122)	89 ± 59^b^ (63, 114)	90 ± 61 (63, 116)
60°/s (CONT)	103 ± 50 (79, 133)	109 ± 56 (83, 143)	109 ± 56 (83, 143)	106 ± 52 (82, 138)	104 ± 54 (79, 137)	60°/s (CONT)	75 ± 51 (53, 97)	86 ± 49^b^ (65, 107)	77 ± 51 (55, 98)	86 ± 53^b^ (63, 109)	79 ± 53 (57, 102)
180°/s (HEAT)	69 ± 44 (47, 95)	75 ± 48 (52, 104)	73 ± 45 (52, 101)	74 ± 45 (54, 93)	75 ± 48 (53, 104)	180°/s (HEAT)	66 ± 54 (43, 89)	73 ± 57 (48, 98)	66 ± 58 (41, 91)	70 ± 56 (46, 94)	67 ± 59 (41, 92)
180°/s (CONT)	64 ± 38 (45, 86)	71 ± 37 (54, 93)	71 ± 37 (54, 93)	72 ± 40 (52, 95)	74 ± 43 (55, 98)	180°/s (CONT)	57 ± 49 (36, 78)	67 ± 45 (48, 86)	64 ± 48 (41, 91)	56 ± 47 (36, 76)	68 ± 50 (46, 89)
300°/s (HEAT)	49 ± 35 (33, 70)	53 ± 40 (35, 77)	56 ± 40 (37, 80)	54 ± 39 (36, 78)	51 ± 42 (32, 75)						
300°/s (CONT)	49 ± 35 (32, 69)	52 ± 33 (36, 71)	54 ± 32 (38, 72)	51 ± 34 (35, 71)	54 ± 35 (38, 75)						

Isotonic contractions – peak velocity (°/s)						Isokinetic contractions – RFD_50_					
25% MVIC (HEAT)	281 ± 59 (278, 326)	286 ± 64 (282, 336)	287 ± 62 (285, 336)	297 ± 64 (291, 347)	280 ± 62 (268, 327)	60°/s (HEAT)	597 ± 738 (347, 847)	815 ± 768 (555, 1074)	819 ± 684 (587, 1051)	750 ± 672 (522, 978)	801 ± 702 (564, 1039)
25% MVIC (CONT)	282 ± 55 (278, 326)	297 ± 56 (286, 335)	297 ± 56 (289, 332)	297 ± 62 (286, 343)	301 ± 55 (295, 341)	60°/s (CONT)	610 ± 540 (427, 793)	644 ± 552 (456, 832)	664 ± 516 (488, 840)	669 ± 498 (500, 837)	687 ± 552 (500, 874)

All participants											
Isokinetic contractions – peak torque (N m)						Isokinetic contractions – early force production (N m)					
60°/s (HEAT)	150 ± 53 (132, 173)	154 ± 58 (134, 178)	155 ± 58 (134, 178)	152 ± 56 (133, 175)	151 ± 57 (131, 159)	60°/s (HEAT)^c^	98 ± 54 (82, 113)	109 ± 58^a^, ^b^ (92, 126)	114 ± 56^a^, ^b^ (97, 130)	109 ± 55^a^, ^b^ (93, 126)	110 ± 58^b^ (93, 127)
60°/s (CONT)	140 ± 46 (123, 158)	143 ± 52 (125, 164)	142 ± 52 (124, 163)	139 ± 49 (122, 158)	138 ± 51 (121, 159)	60°/s (CONT)	97 ± 48 (83, 111)	102 ± 34 (89, 116)	99 ± 35 (85, 113)	102 ± 37 (87, 116)	100 ± 37 (85, 115)
180°/s (HEAT)	101 ± 41 (86, 117)	109 ± 45^a^, ^b^ (94, 127)	109 ± 42 ^a^ (94, 126)	108 ± 42^a^ (94, 125)	109 ± 44 (94, 127)	180°/s (HEAT)^c^	93 ± 50 (102, 137)	101 ± 53^b^ (106, 141)	99 ± 54^b^ (108, 145)	100 ± 52^b^ (107, 140)	99 ± 55^b^ (106, 144)
180°/s (CONT)	99 ± 35 (86, 112)	102 ± 35 (90, 116)	102 ± 34 (90, 115)	102 ± 38 (89, 116)	103 ± 39 (91, 119)	180°/s (CONT)	88 ± 46 (40, 83)	93 ± 42 (48, 91)	93 ± 45 (43, 87)	87 ± 44 (43, 83)	95 ± 47 (44, 90)
300°/s (HEAT)	78 ± 32 (66, 90)	84 ± 37^a^, ^b^ (71, 99)	85 ± 37^a^, ^b^ (72, 100)	84 ± 36^a^, ^b^ (72, 99)	81 ± 38 (68, 96)						
300°/s (CONT)	78 ± 32 (66, 90)	79 ± 30 (68, 91)	79 ± 30 (68, 90)	77 ± 31 (66, 89)	79 ± 32 (68, 92)						

Isotonic contractions – peak velocity (°/s)						Isokinetic contractions – RFD_50_					
25% MVIC (HEAT)	342 ± 41 (337, 368)	342 ± 42 (336, 371)	344 ± 41 (338, 371)	349 ± 42 (342, 378)	339 ± 41 (329, 376)	60°/s (HEAT)	792 ± 492 (625, 960)	1022 ± 516^a^, ^b^ (847, 1196)	1057 ± 462^a^, ^b^ (901, 1213)	1002 ± 450^a^, ^b^ (850, 1156)	1015 ± 468^a^, ^b^ (856, 1175)
25% MVIC (CONT)	337 ± 41 (331, 360)	345 ± 40 (337, 368)	344 ± 41 (337, 368)	344 ± 42 (334, 372)	348 ± 40 (342, 372)	60°/s (CONT)	857 ± 360 (735, 980)	898 ± 372 (772, 1024)	910 ± 348 (793, 1029)	898 ± 336 (785, 1012)	915 ± 372 (790, 1041)

^a^ *P* < 0.05 versus control at corresponding time point.

^b^ *P* < 0.05 versus baseline (0 min).

^c^ Main effect for difference between conditions, *P* < 0.05.

^d^ Main effect for difference between visits, *P* < 0.05.

**FIGURE 4:**
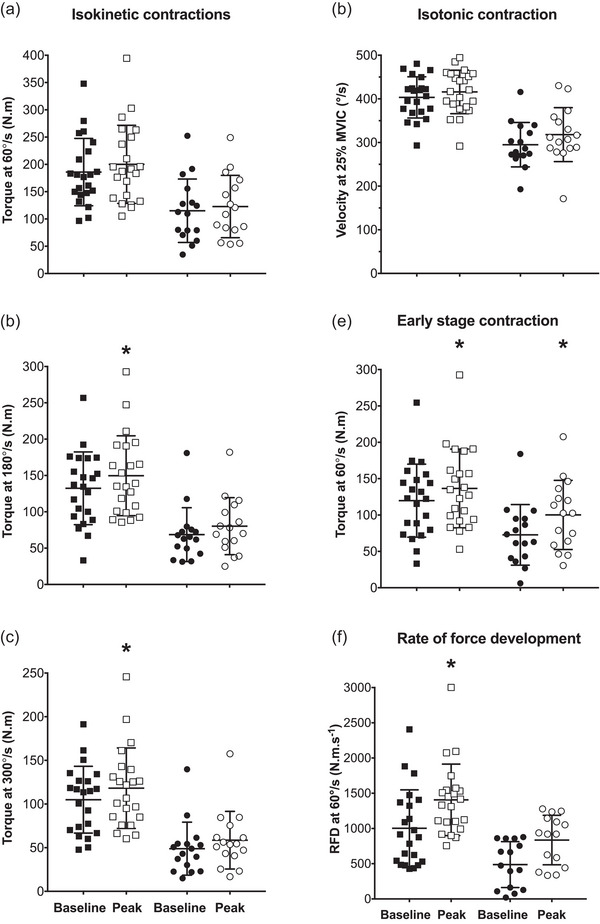
Peak torque production across 60°/s (a), 180°/s (b), 300°/s (c) and maximum isotonic velocity (d), early force across 60°/s (e) and rate of force development in 60°/s (f) at baseline (filled symbols) and peak change (open symbols) following 90 min of passive thigh heating (HEAT). Squares represent YOUNGER group and circles represent OLDER group. *Significant difference from baseline within group (*P* < 0.05).

### 3.4 Rate of force development, early stage force production and surface EMG following upper thigh muscle hyperthermia

When comparing the effect of heating on RFD_50_, the rate of torque development during 60°/s contractions differed between conditions (*F*_(1,34)_ = 5.1, *P* = 0.03, η_p_^2^ = 0.60), whereby the heated leg produced 82 ± 120 N m s^−1^. Differences were seen for the main effect of time (*F*_(4,136)_ = 11.1, *P* ≤ 0.001, η_p_^2^ = 0.99) with an increase in RFD_50_ observed from baseline throughout the 120 min protocol. Condition × time differed (*F*_(4,136)_ = 6.0, *P* ≤ 0.001, η_p_^2^ = 0.96) whereby HEAT saw an increase from baseline of +229 ± 210 N m s^−1^ (+29 ± 26%) after 30 min, +265 ± 282 N m s^−1^ (+33 ± 31%) after 60 min, +211 ± 282 N m s^−1^ (+27 ± 36%) after 90 min and +223 ± 306 N m s^−1^ (+25 ± 34%) 30 min after heating had ceased. No significant changes were observed in CONT from baseline throughout the protocol. No significant differences were observed at baseline between HEAT and CONT; however, after 30 min of heating, Heat was +99 ± 348 N m s^−1^ higher and remained at this difference throughout the protocol.

When comparing the effect of heating on EFP_60_, torque during 60°/s contractions differed between conditions (*F*_(1,35)_ = 9.7, *P* = 0.004, η_p_^2^ = 0.22) whereby the heated leg produced +8 ± 15 N m (8 ± 15%) more force. Differences were seen for the main effect of time (*F*_(4,140)_ = 6.9, *P* ≤ 0.001, η_p_^2^ = 0.15) with an increase in EFP_60_ peak torque observed from baseline throughout the 120 min protocol. Condition × time differed (*F*_(4,140)_ = 5.6, *P* ≤ 0.001, η_p_^2^ = 0.14) whereby HEAT saw an increase of +15 ± 19 N m (+15 ± 19%) at 60 min, +7 ± 16 N m (+8 ± 18%) at 90 min and +10 ± 21 N m (+10% ± 16%) at 120 min when compared to CONT. HEAT also differed from baseline by +12 N m from 30 min onwards (+12 ± 16%). CONT saw no changes. An interaction effect of condition × time × group was observed (*F*_(4,140)_ = 2.8, *P* = 0.043, η_p_^2^ = 0.78); YOUNGER saw a significant increases in HEAT when compared to CONT of +10 ± 26 N m (+8 ± 20%) at 60, 90 and 120 min whilst OLDER saw a significant increase in HEAT when compared to CONT of +20 ± 19 N m (+21 ± 20%) at 60 min but not at 90 min (+3 ± 16 N m) and at 120 min (+10 ± 21 N m). When compared to baseline, HEAT in YOUNGER was significantly improved by +11 ± 16 N m (+8 ± 12%) at 60 min, with OLDER improved by +20 ± 17 N m (+21 ± 18%) at 60 min and +13 ± 17 N m (+15 ± 20%) at 90 min. CONT increased from baseline by 10 ± 14 N m (+15 ± 21%) at 30 and 90 min. Differences were seen at EFP_180_ when observing condition (*F*_(1,35)_ = 8.5, *P* = 0.006, η_p_^2^ = 0.20) where a +7 ± 10 N m (+12 ± 17%) difference was observed between HEAT and CONT. The main effect of time also differed (*F*_(4,140)_ = 3.0, *P* = 0.039, η_p_^2^ = 0.08); a significant increase from baseline was seen at 30 min (+6 ± 9 N m; +8 ± 12%). No significant differences were observed between HEAT and CONT. No main or interaction effects were observed upon investigating EMG results. Data were not significantly different between conditions at any time point and did not differ between YOUNGER and OLDER, with all statistical outcomes related to rate of force development and early stage force production presented in Table A3.

### 3.5 Perceptual measures following upper thigh muscle hyperthermia

Rate of perceived exertion differed when examining the main effects of condition (*F*_(1,35)_ = 12.4, *P* = 0.001, η_p_^2^ = 0.26) and time (*F*_(4,140)_ = 18.0, *P* =  < 0.001, η_p_^2^ = 0.34) and the interaction effects of condition × group (*F*_(1,35)_ = 4.9, *P* = 0.033, η_p_^2^ = 0.12), time × group (*F*_(4,140)_ = 5.4, *P* ≤ 0.001, η_p_^2^ = 0.14) and condition × time × group (*F*_(4,140)_ = 2.6, *P* = 0.043, η_p_^2^ = 0.07). In YOUNGER HEAT and CONT, a significant change from baseline of +2 on the RPE scale was seen from 30 min onwards. OLDER saw no increase from baseline in HEAT or CONT. YOUNGER saw significant differences between HEAT and CONT, with HEAT being −1 AU at every time point compared to CONT. Thermal sensation differed for the main effect of time (*F*_(4,144)_ = 30.8, *P* ≤ 0.001, η_p_^2^ = 0.46) and time × group (*F*_(4,144)_ = 3.6, *P* = 0.008, η_p_^2^ = 0.09, see Table 2). A total of 35% of YOUNGER participants self‐reported that heating made them feel at least ‘moderately better’ in terms of readiness for exercise, 50% self‐reported ‘somewhat better’ and 35% self‐reported ‘a little bit better’. In the OLDER group 31% self‐reported that heating made them feel at least ‘quite a bit better’ in terms of readiness for exercise, 56% self‐reported ‘moderately better’, 69% self‐reported ‘somewhat better’ and 88% self‐reported ‘a little bit better’.

## 4 DISCUSSION

This study primarily aimed to assess the effects of 90 min of passive thigh heating on peak torque, EFP and RFD during isokinetic contractions in YOUNGER and OLDER adults. Secondary objectives included evaluating the retention of these effects 30 min post‐heating and examining age‐related differences in perceived impacts of passive heating on muscle function. The passive thigh heating intervention significantly increased *T*_mu_ in HEAT by +5.3°C, which occurred in the absence of a change in any systemic physiological responses in both YOUNGER and OLDER groups. In conjunction with increased *T*_mu_, peak torque increased in HEAT during tests at moderate (+6%) and fast (+8%) contractile speeds when compared to CONT. When exploring between age group differences, at the moderate contractile speed, YOUNGER were the only group significantly changing from baseline by 11%, whilst OLDER saw no significant change at any time point. This outcome may indicate that the thermally mediated mechanism driving change was not activated/present in the OLDER group. When observing RFD_50_, combined data from the YOUNGER and OLDER groups saw a 29% increase from baseline in HEAT. EFP also increased from baseline in HEAT by 9% in the YOUNGER and by 28% in the OLDER groups. In light of this, it is possible that older skeletal muscle may have a greater capacity to enhance EFP than younger tissue following passive heating; however, this supposition requires further consideration and investigation. The reported changes in peak torque at moderate and fast contractile speeds and in EFP in YOUNGER were above the previously reported minimum detectable change (MDC_95_) in 65%, 75% and 95%, with 44%, 38% and 75% of OLDER adults exceeding this threshold (Denny et al., 2025). Interestingly, whilst fast isokinetic contractions improved following HEAT, the fastest/maximal velocity contraction type, that is, isotonic contractions, saw no change at any time point or between conditions. OLDER adults perceived heating to increase their readiness for exercise more than YOUNGER adults, although both considered it beneficial.

### 4.1 Neuromuscular, physiological and perceptual responses to passive thigh heating

The finding of overall positive changes in peak torque in moderate and fast contractions following passive heating aligns with prior experimental findings in young adults (Denny et al., 2025). Peak torque in the heated limb within YOUNGER was significantly enhanced by 8% from baseline after just 30 min of heating, and this was sustained throughout the protocol, whilst the CONT limb saw no improvements from baseline. The OLDER group, however, only reported a non‐significant 7% increase after 30 min. All increases in muscle function displayed large effect sizes (>0.14 η_p_^2^), providing support for the positive impact that passive thigh heating had on peak torque and EFP. Our findings are in broad agreement with prior work examining passive heating that has observed RFD improvements of +26% at 50 ms (Rodrigues et al., 2021) and +48% at 100 ms (Mornas et al., 2022) in young adults. The significant improvements in EFP function observed during heating did not decay 30 min following heating cessation, highlighting that benefits persist beyond the withdrawal of the heating intervention, at least in the short‐term window after heating withdrawal. Whilst this study did not attempt to identify mechanistic underpinnings, the muscular benefits persisting after heating had ceased may be in part caused by retained enhancements in microcirculatory and microvascular function (Brunt et al., 2016), as well as improved endothelium‐dependent vasodilation (Richey et al., 2022). The upper heating duration for which a positive benefit is observed requires identification. In contrast ith the retention of EFP, the observed increases in peak torque had diminished 30 min following heating cessation, drawing parallels to prior work using active warm‐ups (Faulkner et al., 2013). This may indicate the mechanisms associated with this positive outcome are thermally mediated rather than vascular in origin. The absence of significant increases in systemic physiological measures across the protocol, that is, heart rate, *T*_tymp_ and MAP were stable throughout heating, suggests an increased risk of adverse heat‐related events is unlikely to be associated with the intervention. Rating of perceived exertion scores in HEAT were lower than those in CONT and this may be due to the analgesic effect of heating reducing the negative or painful experiences caused by high force contractions (Chabal et al., 2020).

### 4.2 Age‐related differences in the responses to passive thigh heating

Peak torque within YOUNGER adults was significantly enhanced by 8% from baseline after just 30 min of heating. This increase was sustained throughout the protocol whilst the CONT limb saw no improvements from baseline. The OLDER group, however, only reported a non‐significant 7% increase in peak torque (Figure 4). These results suggest that whilst passive heating may offer advantages for peak isokinetic force production in young individuals, the benefits of passive thigh heating on this outcome specifically may be limited or absent in older adults when contextualised against inferential statistical outcomes. Beyond 30 min of heating, examination of the OLDER peak torque responses indicates a more compelling absence of benefit, given relative change diminishes (≤5% thereafter), suggesting there may be an optimal time course for the intervention. The RFD in HEAT saw an increase of 29% from baseline and a difference from CONT of +16% from 30 min onwards. Similarly, both YOUNGER and OLDER groups benefited from heating when examining EFP at the slowest isokinetic contraction speed (EFP_60_). The peak isokinetic torque changes observed in YOUNGER moderate speed were above the calculated minimal detectable change (+11 N m, MDC_95_ = 6 N m) whilst the non‐significant changes of OLDER were lower than MDC_95_. The group mean increases in peak isokinetic, EFP and RFD_50_ are also above the calculated MDC_95_ in moderate and fast contractions (6 and 7 N m, respectively). YOUNGER recorded an 8% increase in EFP_60_ in HEAT from 60 min onwards, whilst OLDER recorded a 20% increase in EFP_60_ in HEAT. Prior work (Denny et al., 2025), displayed a 15% increase in EFP_60_ during passive heating in YOUNGER, which agrees with the outcomes in the OLDER group in the current study. These outcomes may indicate that age does not modulate the improvement in EFP; however, this construct requires further experimental examination. Relatedly, this outcome could indicate that a similar mechanism associated with improved EFP is active in both age groups, and this mechanism may be separate from that which drives increases in peak torque. The greater increase of EFP observed in OLDER may suggest a greater sensitivity or potential to respond to the proposed temperature‐regulated mechanisms associated with EFP_60_, for example, enhanced nerve conduction velocity. This may also explain why OLDER saw increases in the CONT condition, as slight *T*_mu_ increases were also observed in that condition, as an ∼5% increase in conduction velocity has been observed in healthy younger adults for each 1°C increase in *T*_mu_ (Kiernan et al., 2001). Older adults typically display a reduced time to muscle activation and therefore may benefit more greatly from the increases in nerve conduction velocity following passive heating (Thelen et al., 2000). Further research is required to understand if the changes in conduction velocity observed are present in older adults and if those responses are present during maximal knee extension contractions.

Higher RFD_50_ has been positively associated with increased mobility and increased ability to complete daily tasks (Aagaard et al., 1989; Blazevich et al., 2009; Maffiuletti et al., 2016). Whilst muscular weakness is a moderate predictor of fall risk in older adults (Suetta et al., 2004), the ability to reverse a fall and prevent injury is more closely linked to rapid force production, with RFD_50_ a proxy to this functional outcome (Bellumori et al., 2013). Muscular power has been identified as a key determinant of functional performance, more so than muscular strength, for the execution of daily tasks, particularly those requiring rapid and dynamic movements (Bean et al., 2002; Sayers, 2008). Functional tasks such as rising from a chair, climbing stairs and maintaining balance during unexpected perturbations often necessitate the rapid generation of force, rather than merely the ability to sustain or produce high maximal force. This distinction underscores the practical importance of muscular power in maintaining independence and reducing fall risk, particularly in older populations (Gray & Paulson, 2014). An individual's RFD_50_ is widely recognised as the main determinant of muscular power, as it reflects the ability of the neuromuscular system to generate force within the critical time frames required for successful task completion (Mclellan et al., 2011). Therefore, the observed benefits in this metric have considerable importance from a translational perspective. In this context, it is imperative to recognise that declines in muscular power with ageing are more pronounced than those observed in maximal strength (Macaluso & de Vito, 2004). This pronounced decline in muscular power has been termed powerpenia (Freitas et al., 2024). Unlike sarcopenia, which focuses on the loss of muscle mass, powerpenia specifically impairs rapid force production required for tasks such as rising from a chair, climbing stairs or preventing a fall. It typically precedes declines in muscle strength and mass, making it a sensitive early marker of functional impairment in older adults (Freitas et al., 2024). This disparity highlights the need to prioritise strategies that specifically target power development in older adults, for example, increasing *T*_mu_, as peak muscular force development alone may fail to address the time‐sensitive demands of functional tasks (Freitas et al., 2024).

Current research suggests multiple mechanisms may be responsible for improved muscle function following passive heating, including an increase in localised blood flow into and out of the heated muscle (Chiesa et al., 2015; Watanabe et al., 2024), improved myofibrillar calcium handling (Kobayashi et al., 2005), altered muscle–tendon stiffness (Rodrigues et al., 2022), optimised penetration angle (Eng et al., 2018), and increases in ATP turnover and muscle fibre conduction velocity (Gray et al., 2006). The outcomes of this experiment, specifically the divergent responses between our groups, indicate that these proposed mechanisms underpinning increased peak and early isokinetic torque following passive heating may not be similarly inducible in the muscle of older adults in the way that they appear to be in experiments involving only younger adults. This study did not seek to extensively understand the fundamental mechanisms associated with muscle force enhancement following passive heating, but a notable finding of no change in peak surface EMG may suggest that the observed increases in force production are not due to an increased maximal neural drive at the working muscle (Aagaard et al., 2002) and indicates that an alteration in local muscle structure/function is more likely to be responsible for the change in isokinetic performance (Fitts et al., 1991). Mechanisms underpinning differential age‐specific outcomes in response to passive heating require extensive further examination and are likely influenced by the multiple and complex alterations in skeletal muscle anatomy and physiology that occur with advanced age. One such example is the theory that an increase in intramuscular fluid both stiffens the muscle and re‐orders the angle of the muscle fibres to be more optimal for exercise (Eng et al., 2018). Whilst present in younger adults, this adaptive response may be less applicable to older adults who generally have higher deposits of intramuscular fat (Pinel et al., 2021), which is compressible, and so the muscle does not deform into the theorised optimum. The OLDER group within this study had a ∼10% higher body fat percentage than YOUNGER, and whilst intramuscular fat was not measured directly, those with a higher overall body fat percentage have a greater absolute fat mass, and given ageing results in fat redistribution, it is likely greater fat deposits reside in this tissue in OLDER (Li et al., 2022). Ageing also leads to impairments in the sarcoplasmic reticulum calcium ATPase (SERCA) pump, which then slows the release of calcium into the working muscle (Miljkovic et al., 2015) and the subsequent calcium reuptake (Carmeli et al., 2002; Hunter et al., 1999). In young healthy adults, the SERCA pump is stimulated during skeletal muscle hyperthermia (Davies & Young, 1983), thereby increasing reuptake of calcium and reducing muscle half relaxation time (Rodrigues et al., 2021). Given that the SERCA pump is vulnerable to numerous muscle pathologies associated with general ageing (Xu & van Remmen, 2021), this may, in turn, lead to a blunted ability for stimulation following passive heating. This line of reasoning also requires direct research to either confirm or rebut. Ageing often leads to greater motor neuron function variability (Welsh et al., 2007) and a decrease in nerve conduction velocity (Rivner et al., 2001); however, in this study, it is possible that a similar neural effect was eventually observed in both YOUNGER and OLDER, resulting in the equivalent EFP_60_ changes across groups. The degradation of the neural pathways may, however, explain why 60 min of heating was required in the OLDER group, whilst only 30 min was required to see significant benefits in the YOUNGER group. Whilst this may be a temporal factor, it is worth considering that it may be temperature‐dependent dependant whereby older adults require higher temperatures to elicit the same outcome. Further mechanistic work examining the influence of incremental *T*_mu_ on contractile function across the lifespan is needed. When observing perceptual data, it is worth noting that the OLDER group perceived a greater sense of readiness for exercise following passive heating than the YOUNGER group. Whilst it is unclear how this relates to the physiology of muscle function, improving perceptions of readiness for exercise may bolster motivation and confidence, which could improve exercise participation and adherence (Lee et al., 2008). The reported enhancement in ‘readiness for exercise’ underscores the potential of localised heating as a preparatory strategy for physical activity that does not undesirably perturb systemic physiology.

### 4.3 Experimental implications and directions for future research

Given the relative infancy of the field, there are numerous opportunities for further work. Whilst peak muscle activation was not changed in this study, due to the technological limitations of the equipment used, it is not possible to determine the impact of increased nerve conduction rate on EFP following passive heating (Todnem et al., 1989). Time‐syncing the EMG sensors to the isokinetic dynamometer would have allowed for an observation of muscle activation during the early stages of force production and should be implemented in future experimental studies. This study is also somewhat limited in its analysis of RFD_50_ due to the 100 Hz export rate from the dynamometer device. Whilst participants were instructed to maximally contract during the exercise, this was not confirmed by any maximal EMG baselines acquired through external muscle stimulation in this study, nor was an assessment of voluntary activation made during each contraction. It is also acknowledged that *T*_mu_ measurements were taken without ultrasound guidance; therefore, it cannot be guaranteed that all *T*_mu_ measurements were conducted at the same muscle depth between each participant (Rodrigues et al., 2024). Previous research on prolonged passive heating reported decreases in *T*_mu_ in the control condition, likely due to the absence of physical activity (Gibson et al., 2023). However, in this study, *T*_mu_ in the control condition increased modestly, likely because of the contractions performed, and then plateaued. It can be postulated that passive heating benefits could have an even more pronounced effect when compared to completely inactive, normothermic muscles. We therefore suggest that research investigating true ‘cold starts’ be undertaken. A direct measure of deep body temperature, for example, rectal or oesophageal temperature, would give further confidence that the null responses observed in *T*_tymp_ were valid. Previous work does, however, support the absence of meaningful increases in core temperature using a 90‐min single limb heating design (Gibson et al., 2023; Koch Esteves et al., 2021; Watanabe et al., 2024). Further research is required to understand the mechanisms behind the observed change in heated muscle in both age groups. Research should also expand into more functional tasks, exploring how heating affects muscle contractions and muscular fatigue over longer time periods than observed in the present study. The effect of heat acclimation on muscle function increases following passive heating also requires investigation. The potential interactive mechanisms between heat acclimation and passive heating on neuromuscular performance remain unclear (Tyler et al., 2016). An extension of this is the need for further investigation into how local versus systemic heating affects maximal muscle function when applied acutely and repeatedly in both males and females. Prospective work in pre‐menopausal female participants should be conducted in accordance with best practice guidelines, that is, recording or controlling for menstrual cycle phase and contraceptive use (Smith et al., 2022). Future research should also endeavour to examine how passive heating may transfer to commonly used functional tests, such as the timed up and go or sit‐to‐stand test. Further research should also investigate how heating may potentially enhance static and dynamic balance in older adults, as temperature‐mediated improvements in proprioception may reduce the risk of falls.

### 4.4 Conclusion

In summary, passive thigh heating increased muscle temperature by >5°C during the protocol, whilst tympanic temperature and other systemic physiology remained unchanged. Peak isokinetic force in younger adults improved during heating at moderate (+8%) and in younger and older adults during fast (+10%) contractile speeds. The rate of force development during slow isokinetic contractions increased from baseline by 29% when younger and older adult data were combined. Early force production during slow isokinetic contractions increased from baseline in younger (+13%) and older adults (+28%) during passive heating. Whilst there are observable differences in the type of responses of younger and older adults to passive heating, taken together, heating can be considered as a beneficial tool to improve muscle function for younger and older adults.

## AUTHOR CONTRIBUTIONS

Conceptualization: Desmond Denny, Daniel C. Low and Oliver R. Gibson. Data curation: Desmond Denny and Oliver R. Gibson. Formal analysis: Desmond Denny, Daniel C. Low and Oliver R. Gibson. Investigation: Desmond Denny, Daniel C. Low and Oliver R. Gibson. Methodology: Desmond Denny, Daniel C. Low and Oliver R. Gibson. Visualisation: Desmond Denny and Oliver R. Gibson. Writing (original draft preparation): Desmond Denny. Writing (review and editing): Desmond Denny, Daniel C. Low and Oliver R. Gibson. All authors have read and approved the final version of this manuscript and agree to be accountable for all aspects of the work in ensuring that questions related to the accuracy or integrity of any part of the work are appropriately investigated and resolved. All persons designated as authors qualify for authorship, and all those who qualify for authorship are listed.

## CONFLICT OF INTEREST

None declared.

## FUNDING INFORMATION

None.

## Data Availability

The datasets used and/or analysed during the current study are available at Figshare: https://figshare.com/s/b1493067f473fa66a718 (DOI: https://doi.org/10.17633/rd.brunel.28303022).

## 1

Table A1, Table A2, Table A3

**TABLE A1:** Full ANOVA results for physiological variables.

		Heart rate	MAP	Tympanic	T. sensation
Time	Mauchly's	<0.001	0.002	<0.001	<0.001
	*F*	2.682	6.057	1.050	30.796
	*P*	0.034	<0.001	0.384	<0.001
	ES (partial η^2^)	0.071	0.148	0.029	0.461
	Observed power	0.589	0.984	0.180	1.000
Group × Time	Mauchly's				
	*F*	1.038	0.727	1.356	3.566
	*P*	0.390	0.575	0.252	0.008
	ES (partial η^2^)	0.029	0.020	0.037	0.090
	Observed power	0.321	0.230	0.415	0.861

**TABLE A2:** Full SPSS ANOVA results table for peak torque and peak velocity.

		Isokinetic torque at 60°/s	Isokinetic torque at 180°/s	Isokinetic torque at 300°/s	Isotonic velocity at 25% MVC
Condition	Mauchly's				
	*F*	13.293	6.675	5.862	0.025
	*P*	<.001	0.014	0.021	0.874
	η_p_^2^	0.27	0.156	0.140	0.001
	power	0.944	0.710	0.654	0.053
Condition × Group	Mauchly's				
	*F*	0.272	1.729	3.946	2.572
	*P*	0.605	0.197	0.055	0.117
	ES (partial η^2^)	0.007	0.046	0.099	0.065
	Observed power	0.08	0.249	0.489	0.346
Time	Mauchly's	0.015	<0.001	<0.001	0.009
	*F*	1.59	5.771	2.793	0.987
	*P*	0.18	0.004	0.064	0.401
	ES (partial η^2^)	0.042	0.138	0.072	0.026
	Observed power	0.481	0.874	0.550	0.262
Time × Group	Mauchly's				
	*F*	0.395	0.441	0.297	1.805
	*P*	0.812	0.800	0.880	0.131
	ES (partial η^2^)	0.011	0.011	0.008	0.047
	Observed power	0.139	0.144	0.115	0.540
Condition × Time	Mauchly's	0.003	<0.001	0.030	0.332
	*F*	0.125	1.185	3.541	1.690
	*P*	0.973	0.320	0.015	0.155
	ES (partial η^2^)	0.003	0.032	0.090	0.044
	Observed power	0.072	0.365	0.858	0.509
Condition × Time × Group	Mauchly's				
	*F*	0.979	3.084	1.611	0.435
	*P*	0.406	0.018	0.175	0.783
	ES (partial η^2^)	0.026	0.79	0.043	0.012
	Observed power	0.261	0.800	0.487	0.150

**TABLE A3:** Full ANOVA results table for early force production and rate of force development.

		Isokinetic torque at 0.18s during 60°/s	Isokinetic torque at 0.18s during 180°/s	Rate of force production at 50ms
Condition	Mauchly's			
	*F*	9.715	8.513	5.126
	*P*	0.004	0.006	0.030
	ES (partial η^2^)	0.217	0.196	0.131
	Observed power	0.858	0.810	0.595
Condition × Group	Mauchly's			
	*F*	0.033	0.275	0.306
	*P*	0.857	0.603	0.584
	ES (partial η^2^)	0.001	0.008	0.009
	Observed power	0.054	0.080	0.084
Time	Mauchly's	<0.001	<0.001	0.004
	*F*	6.307	3.040	11.101
	*P*	0.001	0.039	<0.001
	ES (partial η^2^)	0.153	0.080	0.246
	Observed power	0.937	0.660	1.000
Time × Group	Mauchly's			
	*F*	1.323	0.864	0.292
	*P*	0.264	0.487	0.883
	ES (partial η^2^)	0.036	0.024	0.009
	Observed power	0.405	0.270	0.113
Condition × Time	Mauchly's	0.018	<0.001	0.047
	*F*	5.632	1.358	6.015
	*P*	<0.001	0.252	<0.001
	ES (partial η^2^)	0.139	0.037	0.150
	Observed power	0.976	0.415	0.962
Condition × Time × Group	Mauchly's			
	*F*	2.814	1.613	0.800
	*P*	0.028	0.174	0.527
	ES (partial η^2^)	0.074	0.044	0.023
	Observed power	0.757	0.487	0.251
